# Hospital at Home for Intrathecal Pump Refills: A Prospective Effectiveness, Safety and Feasibility Study

**DOI:** 10.3390/jcm10225353

**Published:** 2021-11-17

**Authors:** Lisa Goudman, Ann De Smedt, René Huygens, Marc Noppen, Maria Vanschoenwinkel, Samar M. Hatem, Maarten Moens

**Affiliations:** 1 Department of Neurosurgery, Universitair Ziekenhuis Brussel, Laarbeeklaan 101, 1090 Brussels, Belgium; maarten.moens@uzbrussel.be; 2 STIMULUS Consortium (reSearch and TeachIng neuroModULation Uz bruSsel), Vrije Universiteit Brussel, Laarbeeklaan 103, 1090 Brussels, Belgium; Ann.DeSmedt@uzbrussel.be (A.D.S.); Samar.Hatem@uzbrussel.be (S.M.H.); 3 Center for Neurosciences (C4N), Vrije Universiteit Brussel, Laarbeeklaan 103, 1090 Brussels, Belgium; 4 Pain in Motion (PAIN) Research Group, Department of Physiotherapy, Human Physiology and Anatomy, Faculty of Physical Education and Physiotherapy, Vrije Universiteit Brussel, Laarbeeklaan 103, 1090 Brussels, Belgium; 5 Research Foundation—Flanders (FWO), 1090 Brussels, Belgium; 6 Department of Physical Medicine and Rehabilitation, Universitair Ziekenhuis Brussel, Laarbeeklaan 101, 1090 Brussels, Belgium; 7 Nursing Department, Universitair Ziekenhuis Brussel, Laarbeeklaan 101, 1090 Brussels, Belgium; rene.huygens@uzbrussel.be (R.H.); Maria.Vanschoenwinkel@uzbrussel.be (M.V.); 8 Chief Executive Officer, Universitair Ziekenhuis Brussel, Laarbeeklaan 101, 1090 Brussels, Belgium; Marc.Noppen@uzbrussel.be; 9 Department of Radiology, Universitair Ziekenhuis Brussel, Laarbeeklaan 101, 1090 Brussels, Belgium

**Keywords:** hospital at home, intrathecal drug delivery, pilot study, neuromodulation, pump refill

## Abstract

Continuous Intrathecal Drug Delivery through an implanted pump is a well-known therapeutic option for the management of chronic pain and severe disabling spasticity. To have a successful therapy, pump refills need to be performed at regular time intervals after implantation. In line with the increased applications of Hospital at Home, these refill procedures might be performed at the patient’s home. The aim of this pilot study is to evaluate the feasibility, safety, and effectiveness of intrathecal pump refill procedures at home. Twenty patients were included whereby pump refill procedures were conducted at the patient’s home. To enable contact with the hospital, a video connection was set-up. Tele-ultrasound was used as post-refill verification. All procedures were successfully performed with complete patient satisfaction. Ninety-five percent of the patients felt safe during the procedure, and 95% of the procedures felt safe according to the physician. All patients indicated that they preferred their next refill at home. The median time consumption for the physician/nurse at the patient’s home was 26 min and for the researcher at the hospital 15 min. In light of quality enhancement programs and personalized care, it is important to continue urgent pain management procedures in a safe way, even during a pandemic.

## 1. Introduction

The coronavirus disease 2019 (COVID-19) outbreak, declared as a pandemic by the World Health Organization (WHO) on 11 March 2020 [1], is still a serious global public health concern. This disease is caused by a novel coronavirus which was first discovered in Wuhan, China in December 2019 and later spread rapidly throughout the world [2]. In many countries, public health measures have been implemented in response to the pandemic, such as limitations on the number of people that may gather in public [3]. Moreover, health authorities have temporarily suspended nonessential outpatient consultations and elective health procedures [3,4]. To assure continuity of care for chronic patients during the COVID-19 pandemic, telehealth has been suggested, which is defined as the delivery and facilitation of health and health-related services including medical care, education, health information services, and self-care via telecommunications and digital communication technologies, including digital communication technology, live video conferencing, remote patient monitoring or mobile health apps [4,5]. During the last decade, telemedicine, i.e., the practice of medicine via remote means [5], has already been successfully applied in chronic pain management [6,7,8,9,10].

Intrathecal pump therapy is considered effective for reducing spasticity in patients with neurological disorders, especially when spasticity interferes with their comfort or function and for managing chronic pain [11,12]. After pump implantation, the most commonly performed postoperative “maintenance” procedure is the drug refill, consisting of an aseptic access to the pump reservoir, emptying residual volume, and filling the reservoir with new medication [13]. Successful therapy thus requires continuous input from specialists; in particular, pump refills need to be performed at regular time intervals [14]. Delayed pump refill can cause serious withdrawal syndrome and in the case of baclofen life-threatening events may occur such as rhabdomyolysis, seizure, coma and even death [15]. Thus, intrathecal pump refills and intrathecal pump malfunctioning have been allocated as urgent patient procedures during the COVID-19 pandemic [16]. To limit the risk of obtaining COVID-19 infection at the hospital setting on the one hand and to overcome the often inconvenient transportation to the hospital for severely immobilized patients, pump refill procedures could be performed in the home-setting [17]. In The Netherlands, this approach has already been evaluated with a retrospective analysis, revealing positive results regarding efficacy and safety [17]. Therefore, the aim of this pilot study is to prospectively evaluate the feasibility, safety and effectiveness of intrathecal pump refill procedures at home. It is our hypothesis that refills at home will be safe, feasible and preferred by patients, with proven effectiveness.

## 2. Materials and Methods

### 2.1. Participants

In this study, male and female patients (+18 years old) who received intrathecal drug delivery (IDD) through an implanted pump (SynchroMed II, Medtronic Inc., Minneapolis, MN, USA) were recruited from the department of Neurosurgery of Universitair Ziekenhuis Brussels. Continuous IDD is a common method for the management of chronic pain and severe disabling spasticity. Only patients with a stabilized dose for at least 3 months [17] were eligible. Twenty consecutive patients who were scheduled for a refill were asked to participate in this study. This sample size was deemed sufficient to evaluate the aims of this pilot study (feasibility, safety and effectiveness), and is conducted in a setting which is representative for the target study population [18].

The study protocol was approved by the central ethics committee of Universitair Ziekenhuis Brussels (B.U.N. 1432021000501) on 7 July 2021. The study was registered on clinicaltrials.gov (NCT05015933). All patients provided written informed consent before participation. The study was conducted according to the revised Declaration of Helsinki (1998).

### 2.2. Protocol

This was a prospective pilot study investigating the feasibility, safety and effectiveness of intrathecal pump refill procedures at home, consisting of a single study visit. Patients who provided written informed consent to participate in this study agreed to have one pump refill at their home instead of at the hospital. All patients who consented to participate were contacted the day before the refill to arrange practicalities. The study team consisted of a trained nurse, the physician that is normally conducting the refill procedure, and a researcher at the hospital (i.e., teleconsultant). The nurse performed the refill procedures, under the supervision of the physician. Thus, both the nurse and physician were present at the home of the patient, while the researcher was present remotely.

In the hospital, a sealed box was prepared at the hospital pharmacy for every patient the day before the refill procedure in which the required refill medication was stored at room temperature. The prescription and medication label were double-checked by the hospital pharmacist and refill team.

**Figure 1:**
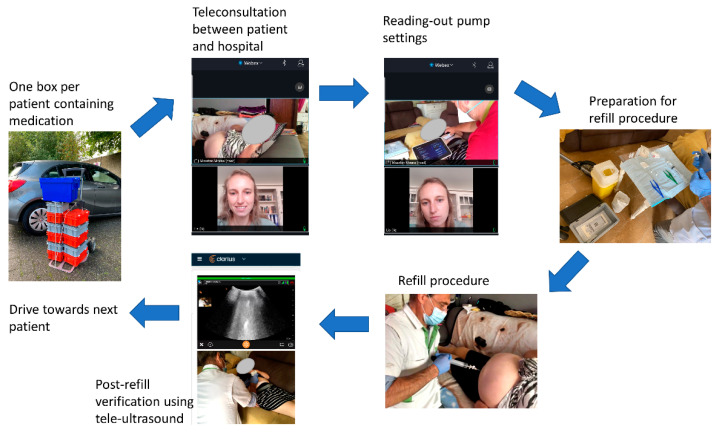
Study flow chart to refill intrathecal pumps at the patient’s home.

At first, when arriving at the patient’s home, the teleconsultation was set-up to ensure that the researcher at the hospital could hear and see the full procedure. This video and audio connection was set-up with WebEx (Cisco Systems, Milpitas, CA, USA) via a secured link. This connection provided the patient the opportunity to communicate with the researcher at the hospital, as well as remote visual control of the full procedure. Then, the trained nurse read out the pump settings and performed the sterile refill procedure, under supervision of the physician and the remote researcher (i.e., teleconsultant). In case the procedure could not be performed in a clean and sterile way, this would have resulted in a failure of the refill procedure and the patient would have been scheduled for a refill at the hospital. After the refill, a verification (i.e., post-refill evaluation) was performed using an ultrasound (Clarius C3 Scanner with needle enhancement and telemedicine, Vancouver, Canada) to ensure that the pump was correctly refilled without the presence of subcutaneous drug injection. After this confirmation, the connection was terminated. Questionnaires were filled in immediately after the termination of the connection. During the patient’s visits, the current COVID-19 regulations were strictly followed. Figure 1 is presenting the study flow chart.

### 2.3. Questionnaires

#### 2.3.1. Primary Outcome Measurements: Effectiveness and Safety

Effectiveness and safety (patients and physician) were the primary outcome measurements, leading to multiple (i.e., 4) primary outcome measurements. The primary effectiveness outcome for patients was patient satisfaction, measured with a seven-point Likert scale asking the patient to rate the overall level of satisfaction with the refill at home. Seven-point response scales were preferred due to a proper balance between ease of use and quick to scan [19], optimized reliability [19,20], good criterion validity and discriminating power [19] and less interpolation compared to five-point scales [21]. The primary effectiveness outcome for physicians was whether the pump refill was successful. This was questioned with a binary variable to indicate success or failure of this intervention. Both patients and the physician rated the overall level of safety with a seven-point Likert scale. The outcome measurements for the physician were completed by the physician, however, in shared agreement with the trained nurse since both healthcare providers were present at the home of the patient.

#### 2.3.2. Secondary Outcome Measurements: Safety

Several secondary outcome measurements were used in this study. Environmental safety was measured using an open question in which the physician indicated whether there were any situations that were not safe. Additionally, an open question was posed to evaluate whether the procedure could be performed in a clean and sterile way. After the refill was performed, an additional safety measure was incorporated with an ultrasound to evaluate that there were no subcutaneous collections (i.e., no presence of subcutaneous drug injection). All adverse events, serious adverse events and procedure complications were systematically reported by the physician during the study visit. Between two and twelve hours after the refill at home, the researcher at the hospital telephonically contacted all patients to evaluate the occurrence of adverse events.

#### 2.3.3. Secondary Outcome Measurements: Feasibility

Feasibility was scored with a seven-point Likert scale asking the patient and the physician to separately rate the overall level of feasibility with the refill at home.

The quality of the WebEx connection was evaluated by the physician and the researcher in the hospital. Both the quality of the audio and the video were scored using three different Likert-scales, namely (1) quality of audio, (2) quality of video and (3) overall quality of the teleconsultation [22]. Any issues with hardware, software, connectivity, or safety were systematically recorded.

Additionally, the time needed for the procedure was recorded by the physician and the researcher at the hospital. For the physician, the time from entering the house of the patient until the time that the team has left the patient was recorded. For the researcher at the hospital, the time from the beginning until the end of the call was evaluated.

#### 2.3.4. Secondary Outcome Measurements: Patient Preference

All patients were asked to indicate whether they prefer to have their next refill at home.

Supplementary Material File S1 is providing a detailed description of the outcome measurements.

### 2.4. Statistical Analysis

Questionnaires were filled out in Qualtrics, whereafter data processing and statistical analyses were performed by a statistician using statistical software package R studio version 1.4.1106 (R version 4.0.5, Vienna, Austria). Summary statistics for quantitative variables are presented as mean, standard deviation, 95% confidence interval on the mean or 1st quartile, median, 3rd quartile, number of observations, and number of missing values. For categorical variables, absolute counts (*n*) and percentages (%) of patients with data are presented. Correlation analyses were performed between the download and upload speed and the audio, video and general quality of the teleconsultation with Spearman rank correlation (and corresponding Bonferroni correction). *p*-values of 0.05 or less were considered statistically significant.

## 3. Results

### 3.1. Descriptive Statistics

In total, 20 consecutive patients were included in this prospective pilot study. All refill procedures were performed on 10 August and 11 August 2021. Ten males and 10 females took part in this study. Patients had a mean age of 48 (SD:17.4) years and were implanted on average 8.7 (SD:4.1) years ago. The following medication was administrated through the intrathecal pump therapy: morphine (25%), baclofen (60%), morphine and baclofen (10%) and morphine and clonidine (5%).

### 3.2. Effectiveness and Safety

All patients (100%) indicated that they were completely satisfied with the refill procedure at home. The physician indicated that the procedure to refill the pump was successful for all patients (100%). Ninety-five percent of the patients (*n* = 19) strongly agreed with the statement that the refill procedure at home felt safe and one patient (5%) slightly agreed with this statement. The physician completely agreed with this statement for 30% of the performed refills (*n* = 6), agreed for 65% (*n* = 13) and slightly agreed for 5% (*n* = 1). Since the primary outcome measurements concerning effectiveness and safety are fulfilled, secondary outcome measurements are evaluated as well.

Environmental safety was evaluated with an open question, in which the following factors were raised that made the physician felt unsafe or uncomfortable: low sitting couch (45% (*n* = 9)) and refill while sitting on your knees (5% (*n* = 1)). Four times, the physician indicated that certain elements or situations could potentially compromise the refill in terms of a clean and sterile procedure, namely: low general body care and too small workplace (*n* = 1, 5%), low general body care (*n* = 2, 10%) and chronic tobacco use (*n* = 1, 5%). No adverse events were reported.

### 3.3. Feasibility

The refill procedure at home was feasible according to the patient (100% completely agree, *n* = 20) and the physician (20% completely agree (*n* = 4), 80% agree (80%)).

The overall quality of the teleconsultation was positive (good or very good) for 95% of the procedures according to the physician and the researcher at the hospital. Specific details concerning audio quality, video quality and overall quality are presented in Table 1.

For the physician, a median time of 26 (Q1–Q3: 24.5–29) minutes was measured from entering to leaving the house of the patient. For the researcher at the hospital, the median time from beginning to ending the call was 15 (Q1–Q3: 13.75–17) minutes.

For the teleconsultation, 4G was used to avoid coupling the tablet with the personal Wi-Fi network of the patient each time. In two patients’ homes, the 4G network lacked connection speed. Therefore, the personal Wi-Fi network of those patients was used. The median download speed was 53,243 (Q1–Q3: 13,928–94,922) kilobits per second and the median upload speed was 6120 (Q1–Q3: 958–14,979) kilobits per second. Spearman rank correlations revealed four statistically significant correlations that passed the correction for multiple testing, namely (1) a positive correlation between upload speed and overall quality of the teleconsultation as rated by the physician (rs = 0.71, *p* < 0.001), (2) a positive correlation between upload speed and audio quality of the teleconsultation as rated by the physician (rs = 0.64, *p* = 0.002), (3) a positive correlation between upload speed and video quality of the teleconsultation as rated by the physician (rs = 0.71, *p* < 0.001), and (4) a positive correlation between upload speed and video quality of the teleconsultation as rated by the researcher (rs = 0.60, *p* = 0.005).

After refill, no subcutaneous collections were collected by ultrasound (*n* = 14). In six patients, a real-life ultrasound was unavailable for the researcher at the hospital due to low quality of the 4G network (*n* = 3), Wi-Fi issues at the hospital (*n* = 1), or due to patient’s Wi-Fi usage for telemedicine (*n* = 2). However, the physician who was present at the home of the patient was still able to evaluate the ultrasound.

### 3.4. Patient Preference

All patients (100%) indicated that they preferred to receive their following refill at home.

## 4. Discussion

This study aimed to evaluate the effectiveness, safety and feasibility of intrathecal pump refills at home for the first time with a prospective design. All procedures were successfully performed and all patients were completely satisfied with the refill at their homes. Concerning safety, 95% of the patients were comfortable with this procedure at home, and 95% of the procedures felt safe to perform at home according to the physician. If we specifically evaluate the individual response categories, 95% of the patients completely agreed that the procedure was safe, while the physician completely agreed for 30% of the refills and agreed for 65% of the refills. Similar findings were reported for feasibility, where all patients strongly agreed that the refill procedure at home was feasible compared to the physician who rated completely agree for 20% of the refills and agree for 80% of the refill procedures. This indicated that, overall, patients were more likely to give a higher rating on safety and feasibility compared to the physician. This emphasizes the need for a separate and in parallel evaluation of the opinions of all stakeholders in relation to Hospital at Home applications [23,24]. All patients indicated that they preferred their next refill at home.

In general, and especially since the COVID-19 pandemic, the applications of Hospital at home (i.e., providing acute healthcare in a patient’s home as an alternative to traditional inpatient care) have drastically increased [25,26,27]. In patients with heart failure, a meta-analysis indicated that hospital at home significantly increased time to first readmission and improved health-related quality of life compared to routine hospitalization [28]. Costs of index hospitalization were reduced as well [29,30]. A Cochrane review concerning the effectiveness of home-based end-of-life care revealed a higher likelihood of dying at home compared with usual care (RR 1.31, 95% CI from 1.12 to 1.52) and slightly improved patient satisfaction on the short term, wherefore home-based programs were supported by this review [31]. The economic benefit remains uncertain in systematic reviews [28,32]. Several factors, among which the role of specialists, the possibility of multi-session treatments at home, the localization of the hospital, geographical position of patients and economic status of hospitals should be taken into account to fully evaluate the economic impact of hospital at home in a specific center [33]. A proper economic evaluation to determine the cost-effectiveness as well as a full evaluation of patient-reported outcomes concerning health-related quality of life, emotional and psychological well-being should be the focusses of future studies, with a quantification of the additional cost of performing the refill at home in relation to additional health effects. Both a cost-effectiveness analysis (life-years gained as health effect) as well as a cost-utility analysis (quality-adjusted-life-years as health effect) should be executed [34].

The practical implementation of hospital at home outside a research context is often more difficult to achieve and would require organizational changes [35]. Specifically in this study, several environmental factors were mentioned, namely a low sitting couch (45%) and performing the refill while sitting on your knees (5%). These factors are mainly a drawback for healthcare providers, and not necessarily for the patient, which could serve as a potential explanation why physician ratings on feasibility were somewhat lower than the ratings of the patients (i.e., agree with feasibility compared to completely agree with feasibility). In this study, patients themselves could choose the location of the refill procedure, whereby there was no comparison between the height of the bed, sofa or other furniture, which could be performed beforehand if refills at home are implemented in clinical routine care. Additionally, patients could be informed in advance about the required workplace that is needed to perform the refill in a comfortable setting. Finally, it seems justified to ask patients to maintain a proper general body care on the day of the refill, which could limit the barriers of implementing this approach in clinical practice. Additionally, based on previous literature, the following barriers to the implementation of at-home procedures were identified: regulatory barriers and health system policies, electronic health records not designed for hospital at home, inadequate payment mechanisms, and difficulties with collaborative partnerships and communication with all stakeholders [36]. For refill procedures at home in clinical routines, the treating physician could follow the refill procedure using telemedicine while nurse practitioners or nurses with a specific training (eventually with a specialist in training) could perform the refill procedure (cfr. nurse practitioners (i.e., Master’s Degree in Advanced Nursing Practice) in The Netherlands). Therefore, the quality of the WebEx connection was evaluated as well in this study. As an additional safety measure for the physician in the hospital (when this approach is implemented in clinical practice), an ultrasound could be used as a post-refill verification to ensure that no agents were located subcutaneously. A systematic review concluded that ultrasound images that are acquired in resource-limited settings and transmitted using a telemedical platform to an expert interpreter are of satisfactory quality and value for clinical diagnosis and management (known as “tele-ultrasound”) [37]. The current study used this methodology to test whether a remote person at the hospital could perform the post-refill verification with real-time evaluation using tele-ultrasound. This approach was feasible; however, the 4G network capacity needs to be strong enough to enable tele-ultrasound, as shown in this study. Eventually, the general practitioner of the patient could function as the first point of contact to screen for adverse events during the first hours after the refill. Presumably, the hospital pharmacy would still be responsible for preparing and verifying the provided medication, in line with the electronic patients’ record. Specifically for dose changes in an outpatient setting, it is recommended not to exceed more than 10–15% of the daily dose for safety reasons [38].

Presumably, the main limitations of this study are due to the design of the study. This is a pilot study in a limited number of patients, yet representative for the target population, whereby the feasibility, safety and effectiveness of a pump refill procedure at home was evaluated. No comparison was made with the standard (in hospital) approach. Additionally, the study was only conducted in one center, wherefore a larger comparative multicenter trial is needed with an economical evaluation to evaluate the efficacy of this approach.

## 5. Conclusions

In light of quality enhancement programs, personalized care and especially with the ongoing COVID-19 pandemic, it is important to continue urgent pain management procedures in a safe way, even outside the hospital setting. This study has demonstrated for the first time prospectively that intrathecal pump refill procedures can be performed in a hospital-at-home setting, whereby effectiveness, safety and feasibility have been demonstrated in 20 patients. These findings could be the starting point of a new standard care process in chronic pain management.

## Figures and Tables

**Table 1:** Quality assessment of teleconsultation with a specific focus on audio quality, video quality and overall quality according to the physician who is at the patient’s home and the researcher at the hospital for all patients (*n* = 20).

	Perfect. No Distortion or Noise Discernible.	Speech Easily Understandable. Little Noise or Distortion.	Speech Understandable with Slight Effort. Requires Occasional Repetition due to Noise or Distortion.	Speech Understandable with Considerable Effort. Requires Frequent Repetition due To Noise or Distortion.	Unusable. Speech Present but Not Understandable.
Audio quality	Physician: 15 (75%) Teleconsultant: 7 (35%)	Physician: 4 (20%) Teleconsultant: 10 (50%)	Physician: 1 (5%) Teleconsultant: 1 (5%)	Physician: 0 Teleconsultant: 2 (10%)	Physician: 0 Teleconsultant: 0
	Very good	Good	Barely acceptable	Poor	Very poor
Video quality	Physician: 12 (60%) Teleconsultant: 12 (60%)	Physician: 7 (35%) Teleconsultant: 6 (30%)	Physician: 1 (5%) Teleconsultant: 1 (5%)	Physician: 0 Teleconsultant: 1 (5%)	Physician: 0 Teleconsultant: 0
	Very good	Good	Barely acceptable	Poor	Very poor
Overall quality of the teleconsultation	Physician: 12 (60%) Teleconsultant: 8 (40%)	Physician: 7 (35%) Teleconsultant: 10 (50%)	Physician: 0 Teleconsultant: 1 (5%)	Physician: 1 (5%) Teleconsultant: 1 (5%)	Physician: 0 Teleconsultant: 0

## Data Availability

Data available on motivated request by the author.

## Supplementary Materials

The following are available online at https://www.mdpi.com/article/10.3390/jcm10225353/s1, File S1: Description of the outcome measurements.
