# Supplementary material for: Hospital at Home for Intrathecal Pump Refills: A Prospective Effectiveness, Safety and Feasibility Study
Source: J Clin Med. 2021 Nov 17;10(22):5353. doi: 10.3390/jcm10225353 (PMC8617747; doi:10.3390/jcm10225353)
Supplement: Supplementary file 1 [file jcm-10-05353-s001.zip › jcm-1432765-supplementary.pdf]

Supplementary Material

Patient satisfaction

Please rate the following statement: “How satisfied are you with the refill procedure at home?”

1 – Completely dissatisfied
2 – Mostly dissatisfied
3 – Somewhat dissatisfied
4 – neither satisfied or dissatisfied
5 – Somewhat satisfied
6 – Mostly satisfied
7 – Completely satisfied

Successful refill procedure

The intrathecal pump is successfully refilled.

- Yes
- No, please explain what problems you experienced.

Patient and physician safety

Please rate the following statement: “The refill procedure at home felt safe?”

- Strongly disagree
- Disagree
- Somewhat disagree
- Neutral
- Somewhat Agree
- Agree
- Strongly agree

Environmental safety

Where there any environmental factors that made you felt unsafe or uncomfortable? Please define.

Sterile procedure

Where there any elements, situations, … that potentially compromised the refill in terms of a clean, sterile procedure? Please define.

Patient feasibility

Please rate the following statement: “The refill procedure at home is feasible?”

- Strongly disagree
- Disagree
- Somewhat disagree
- Neutral
- Somewhat Agree
- Agree
- Strongly agree

Likert scale for evaluation of delivered audio quality

Delivered Audio Quality	
**5**	Perfect. No distortion or noise discernible.
**4**	Speech easily understandable. Little noise or distortion.
**3**	Speech understable with slight effort. Requires occasional repetition due to noise or distortion.
**2**	Speech understable with considerable effort. Requires frequent repetition due to noise or distortion.
**1**	Unusable. Speech present but not understable.

Likert scale for evaluation of video quality

Video quality	
**5**	Very good
**4**	Good
**3**	Barely acceptable
**2**	Poor
**1**	Very poor

Likert scale for evaluation of overall teleconsultation quality

Overall quality of the teleconsultation	
**5**	Very good
**4**	Good
**3**	Barely acceptable
**2**	Poor
**1**	Very poor

Patient preference

If I have the choice, then I prefer to have my following refill at home:

- Yes
- No
